# MKRN2 is a novel ubiquitin E3 ligase for the p65 subunit of NF-κB and negatively regulates inflammatory responses

**DOI:** 10.1038/srep46097

**Published:** 2017-04-05

**Authors:** Chanyoung Shin, Yuma Ito, Shota Ichikawa, Makio Tokunaga, Kumiko Sakata-Sogawa, Takashi Tanaka

**Affiliations:** 1 Laboratory for Inflammatory Regulation, RIKEN Center for Integrative Medical Sciences (IMS), RIKEN Research Center for Allergy and Immunology (RCAI), Yokohama, Kanagawa 230-0045, Japan; 2 School of Life Science and Technology, Tokyo Institute of Technology, Yokohama, Kanagawa 226-8501, Japan

## Abstract

Activation of NF-κB transcription factor is strictly regulated to prevent excessive inflammatory responses leading to immunopathology. However, it still remains unclear how NF-κB activation is negatively controlled. The PDZ-LIM domain-containing protein PDLIM2 is a nuclear ubiquitin E3 ligase targeting the p65 subunit of NF-κB for degradation, thus terminating NF-κB-mediated inflammation. Using yeast two-hybrid screening, we sought to isolate PDLIM2-interacting proteins that are critical for suppressing NF-κB signaling. Here we identified MKRN2, a RING finger domain-containing protein that belongs to the makorin ring finger protein gene family, as a novel p65 ubiquitin E3 ligase. MKRN2 bound to p65 and promoted the polyubiquitination and proteasome-dependent degradation of p65 through the MKRN2 RING finger domain, thereby suppressing p65-mediated NF-κB transactivation. Notably, MKRN2 and PDLIM2 synergistically promote polyubiquitination and degradation of p65. Consistently, MKRN2 knockdown in dendritic cells resulted in larger amounts of nuclear p65 and augmented production of proinflammatory cytokines in responses to innate stimuli. These results delineate a novel role of MKRN2 in negatively regulating NF-κB-mediated inflammatory responses, cooperatively with PDLIM2.

For protection from pathogen, nuclear factor κB (NF-κB) is a pivotal transcription factor for expression of cytokines (IL-6, IL-12, TNFα), chemokines, growth factors (G-CSF) and effector enzymes to activate the innate immunity and subsequent acquired immunity mediated by T and B cells^1^,^2^. The NF-κB family is composed of five members of subunits, p65 (also called RelA), RelB, c-Rel, p50 and p52. In the innate immune system, Toll-like receptor (TLR) stimulation leads to IκBα degradation by the proteasome, which allows heterodimers of p65 and p50 to translocate into the nucleus and induce the expression of a series of genes that are involved in the initiation of inflammatory responses. On the other hand, dysregulation of NF-κB, resulting in constitutive activation of NF-κB signaling, causes massive inflammatory damage to the host, which may lead to autoimmune diseases such as rheumatoid arthritis, systemic lupus erythematosus, type 1 diabetes, multiple sclerosis, and inflammatory bowel diseases^3^,^4^. Thus, the systems that negatively regulate NF-κB activation are essential for preventing immunopathology.

By sequestering NF-κB in the cytoplasm thereby preventing its nuclear translocation before cells get fully activated, cytoplasmic IκBα functions as a major inhibitor of TLR signaling. On the other hand, activated NF-κB in the nucleus should be downregulated at the appropriate time points to restore homeostasis^5^. We previously identified PDLIM2 (also known as SLIM or mystique) as a suppressor protein responsible for the termination of p65 activation in the nucleus^6^,^7^,^8^,^9^. PDLIM2 is a nuclear protein containing both PDZ (postsynaptic density 65-discs large-zonula occludens 1) and LIM (abnormal cell lineage 11-isket 1-mechanosensory abnormal 3) domains, and functions as a nuclear ubiquitin E3 ligase targeting the p65 subunit of NF-κB. We have demonstrated that p65 is polyubiquitinated in the nucleus and then translocated into the distinct subnuclear compartment called PML nuclear bodies, which correspond to the insoluble nuclear fraction in Western blot analysis. Since PML nuclear bodies are enriched for components of the 26S proteasome, polyubiquitinated p65 is ultimately degraded by the proteasome in this compartment. PDLIM2 binds to p65 and not only promotes its polyubiquitination in the nucleus through the LIM domain, but also mediates intranuclear targeting of p65 to PML nuclear bodies via PDZ domain. PDLIM2 deficiency results in increased amounts of nuclear p65 protein and augmented production of proinflammatory cytokines in response to TLR stimulation^9^.

Since both PDZ and LIM domains are generally thought of as structures involved in protein-protein interaction^10^, we assume that PDLIM2 controls inflammatory responses through its interaction with other intracellular molecules that are also important for regulating inflammatory signaling. Identification of these factors may help to clarify the details of systems that negatively regulate inflammatory responses. We have therefore used a yeast two-hybrid system to isolate PDLIM2-interacting proteins. From this screen, we isolated MKRN2 (makorin ring finger protein 2, also known as RNF62 or HSPC070)^11^,^12^ as a protein that can bind to PDLIM2 and negatively regulate NF-κB signaling. MKRN2 contains four zinc finger domains and one RING finger domain. RING finger domains are composed of eight conserved cysteine (Cys) or histidine (His) residues and are found in several hundreds of proteins acting as ubiquitin E3 ligases^13^, such as MDM2^14^,^15^ and BRCA1^16^. RING finger domains interact with E2 ubiquitin-conjugating enzymes and help to transfer ubiquitin chains from E2 to the substrate proteins^13^. MKRN2 was therefore predicted to function as a ubiquitin E3 ligase; however, no MKRN2 target proteins have been defined until now. Intriguingly, our results indicate that p65 is one such MKRN2 target protein. Here we demonstrate that MKRN2 interacts with PDLIM2 and promotes polyubiquitination and proteasome-dependent degradation of p65 cooperatively with PLDIM2, thereby negatively regulating NF-κB-mediated inflammatory responses. This study delineates a novel function of MKRN2 as a ubiquitin E3 ligase targeting p65 subunit of NF-κB for degradation to negatively regulate inflammatory responses.

## Results

### MKRN2 is a RING finger domain-containing protein that can bind to PDLIM2

**Figure 1:**
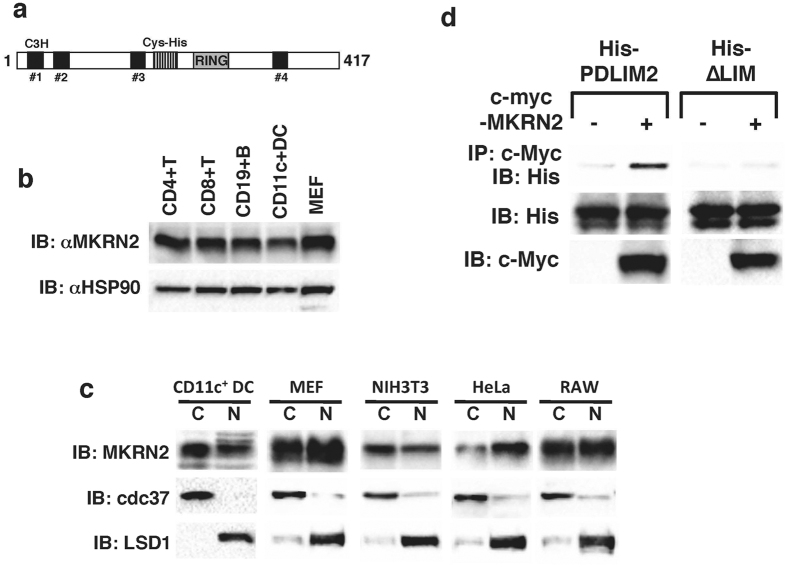
MKRN2 is a RING finger-containing protein that interacts with PDLIM2. (**a**) Schematic diagram of the structure of mouse MKRN2. Black boxes: C3H-type zinc finger domain (C3H), striped box: unique Cys-His motif (Cys-His), gray box: RING finger domain (RING). (**b**) Western blot analysis of MKRN2 expression in primary immune cells and MEFs. Whole cell lysates were subjected to immunoblot (IB) with anti-MKRN2 and HSP90 antibodies. MEF: mouse embryonic fibroblasts. Western blots are representative of at least three independent experiments. (**c**) Subcellular localization of MKRN2 protein. Splenic CD11c^+^ cells, MEF, NIH3T3, HeLa and RAW264.7 cells were fractionated into cytoplasmic (C) and nuclear (N) fractions, which were then analyzed by Western blotting with the MKRN2 antibody. The purity of the fractionations was confirmed by cdc37 (cytoplasm) or LSD1 (nucleus) antibody reactivity. Western blots are representative of at least three independent experiments. (**d**) MKRN2 interacts with PDLIM2 through LIM domain of PDLIM2. 293 T cells were transfected with His-tagged PDLIM2 or the PDLIM2 mutant lacking the LIM domain (∆LIM) along with or without c-Myc-tagged MKRN2. Whole cell extracts were immunoprecipitated with a c-Myc antibody, and immunoblotted with anti-His. Western blots are representative of at least three independent experiments.

To further investigate how inflammatory responses are regulated, we applied a yeast two-hybrid screening strategy to identify molecules that can interact with PDLIM2 and suppress NF-κB signaling. We used a full length murine PDLIM2 as the bait protein. A cDNA library prepared from 18.5 d.p.c. mouse embryos was screened and twenty clones encoding proteins that specifically interacted with the PDLIM2 bait were isolated. One of these clones encoded MKRN2, a member of the MKRN protein gene family^17^,^18^. This family consists of four proteins that all have three C3H-type zinc finger domains, which contain three Cys plus one His, at the N-terminus, followed by an unusual Cys-His motif, a C3HC4-type RING finger domain, and a fourth C3H-zinc finger domain at the C-terminus (Fig. 1a). Although C3H-zinc finger domains have been shown to bind RNA, none of the MKRN family members have been characterized as RNA-binding proteins. But instead, MKRN1 was shown to be a ubiquitin E3 ligase, targeting the telomerase catalytic subunit (hTERT), p53 and p21 for ubiquitin/proteasome-dependent degradation through its RING finger domain^19^,^20^. We therefore predicted that MKRN2 would also possess ubiquitin E3 ligase activity and investigated whether MKRN2 regulates NF-κB-mediated signaling similar to PDLIM2.

A previous study reported that MKRN2 is ubiquitously expressed in hematopoietic cells^21^. We found that MKRN2 is highly expressed in all immune cells tested and in mouse embryonic fibroblasts (MEFs) (Fig. 1b). We next examined the subcellular localization of MKRN2 in primary dendritic cells, MEFs and also in several cell lines. MKRN2 was typically located in both cytoplasm and nucleus, but was predominantly located in the nucleus in some cell-types, such as primary fibroblasts and HeLa cells (Fig. 1c). Finally, to confirm the interaction of MKRN2 and PDLIM2, we transfected human embryonic kidney (HEK) 293 T cells with expression plasmids encoding His-tagged PDLIM2 or the PDLIM2 mutant lacking the LIM domain (∆LIM) together without or with c-Myc-tagged MKRN2. Whole cell extracts were prepared and subjected to immunoprecipitation with a c-Myc antibody and then analyzed by Western blotting with a His antibody. As shown in Fig. 1d, MKRN2 coimmunoprecipitated with wild-type PDLIM2, but not ∆LIM, suggesting that LIM domain of PDLIM2 is essential for the binding between MKRN2 and PDLIM2.

### MKRN2 binds the p65 subunit of NF-κB and negatively regulates NF-κB signaling

**Figure 2:**
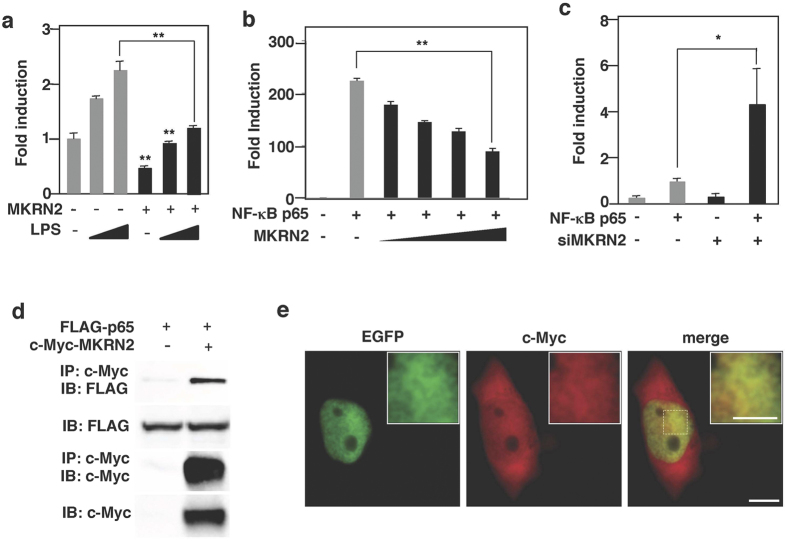
MKRN2 binds to p65 and suppresses NF-κB signaling. (**a**) Luciferase activity in NIH3T3 cells transfected with an ELAM-1 luciferase reporter construct (ELAM-1-luc) along with or without a plasmid encoding MKRN2, then left untreated or treated with LPS for 5 hr. Data are representative of at least three independent experiments. (**b**) Luciferase activity in NIH3T3 cells transfected with an ELAM-1-luc with or without plasmids encoding p65 in the absence or presence of increasing amounts (wedge) of MKRN2. Data are representative of at least three independent experiments and are shown as mean ± SD. **P < 0.01. (**c**) Luciferase activity in NIH3T3 cells first transfected with control or MKRN2-specific siRNA and then with an ELAM-1 luc without or with the plasmid encoding p65. Data are representative of at least three independent experiments and are shown as mean ± SD. *P < 0.05. (**d**) MKRN2 interacts with the p65 subunit of NF-κB. 293 T cells were transfected with a FLAG-tagged-p65 expression plasmid along with or without MKRN2. Whole cell extracts were immunoprecipitated with anti-c-Myc, and immunoblotted with anti-Flag. Western blots are representative of at least three independent experiments. (**e**) Colocalization of MKRN2 with p65 in the nucleus. HeLa cells were transfected with expression plasmids encoding p65 fused with EGFP and c-Myc-tagged MKRN2, then analyzed by fluorescence imaging with EGFP (left) or indirect immunofluorescence imaging with the Myc primary antibody and Alexa Fluor 647-conjugated goat anti-mouse IgG1 (middle). Bar: 10 μm. Inset images are close-ups of the dotted square area. Bar: 5 μm.

Since PDLIM2 negatively regulates TLR-mediated NF-κB signaling^9^, we tested the effect of MKRN2 on TLR-induced, NF-κB-mediated gene activation in a reporter assay. NIH3T3 cells were transfected with a plasmid encoding a luciferase reporter gene regulated by NF-κB. Twenty hours after transfection, cells were left untreated or treated with lipopolysaccharide (LPS) for 5 hr and then assayed for luciferase activity. Stimulation of cells with LPS augmented luciferase activity, whereas expression of MKRN2 potently suppressed transactivation of the LPS-induced luciferase reporter (Fig. 2a). Since MKRN2 interacts with PDLIM2, which targets p65 for inactivation, we next examined the effect of MKRN2 on p65-mediated gene activation in a reporter assay. We cotransfected NIH3T3 cells with p65, to activate the NF-κB luciferase construct, together with or without MKRN2 to test if MKRN2 could inhibit gene activation by p65. Indeed, MKRN2 markedly inhibited p65-induced NF-κB-mediated transactivation of the reporter in a dose-dependent manner (Fig. 2b). Moreover, specific knockdown of MKRN2 by small interfering RNA (siRNA) resulted in a substantial enhancement of p65-mediated NF-κB transactivation (Fig. 2c). We then examined if MKRN2 bound to p65. 293 T cells were transiently transfected with expression plasmids encoding FLAG-tagged p65 with or without c-Myc-tagged MKRN2. MKRN2 was immunoprecipitated with p65 when they were coexpressed (Fig. 2d). To visually confirm this interaction, HeLa cells were transiently transfected with expression plasmids encoding p65 fused with EGFP (enhanced green fluorescent protein) and c-Myc-tagged MKRN2 and imaged by indirect immunofluorescence using epi-fluorescence microscopy. As shown in Fig. 2e, MKRN2 localized together with p65 in the nucleus, where it is noteworthy that they display the same localization pattern. These data suggest that MKRN2 binds to the p65 subunit of NF-κB and inhibits p65-mediated NF-κB activation.

### MKRN2 promotes p65 ubiquitination and degradation through RING finger domain

**Figure 3:**
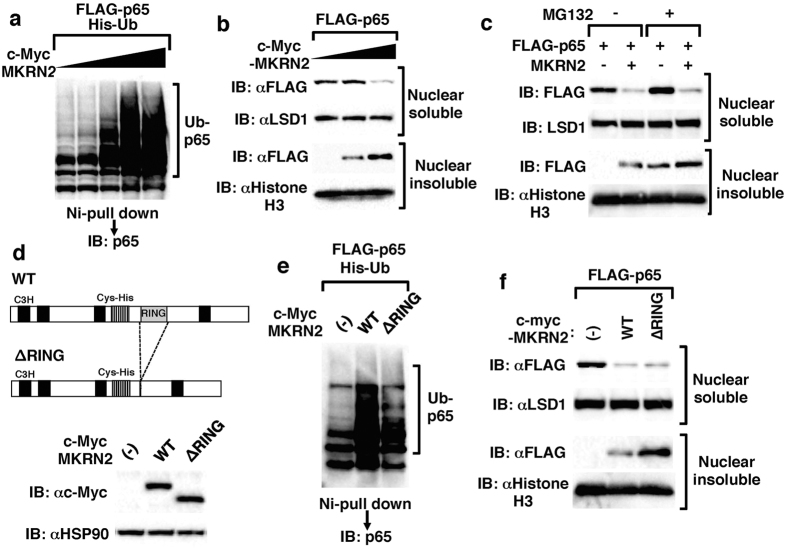
MKRN2 promotes polyubiquitination and degradation of p65. (**a**) Ubiquitination assay for p65 in 293 T cells cotransfected with plasmids encoding His-tagged ubiquitin (His-Ub), FLAG-tagged p65 and increasing amount of MKRN2. His-tagged proteins were purified by Ni-Sepharose beads. Polyubiquitination of p65 was analyzed by anti-p65. (**b**) Effect of MKRN2 on soluble and insoluble nuclear p65 in NIH3T3 cells transfected with plasmids encoding FLAG-tagged p65 and increasing amounts of c-Myc-tagged MKRN2. Cells were subjected to fractionation and analyzed by anti-FLAG antibody. The purity of the fractionations was confirmed by anti-LSD1 (nuclear soluble) or anti-Histone H3 (nuclear insoluble) antibody staining. (**c**) Western blot analysis for soluble and insoluble nuclear p65 in NIH3T3 cells transfected with plasmids encoding p65 without or with MKRN2, then left untreated or treated for 4 h with MG132 (10 µM) and analyzed as in (**b**). (**d**) Schematic diagram of the structure of the MKRN2 mutant lacking the RING finger domain (∆RING). Bottom shows Western blot analysis of NIH3T3 cells transfected with expression plasmids encoding wild-type or ∆RING MKRN2, detected with anti-c-Myc antibody. (**e**) Ubiquitination assay for p65 in 293 T cells cotransfected with plasmids encoding His-tagged ubiquitin (His-Ub), and p65, together without or with wild-type or ∆RING MKRN2 and analyzed as in (**a**). (**f**) Effect of ∆RING MKRN2 on soluble and insoluble nuclear p65 in NIH3T3 cells transfected with plasmids encoding p65, together without or with wild-type or ∆RING MKRN2 and analyzed as in (**b**).

RING finger domains are well known for possessing ubiquitin E3 ligase activity^13^, therefore we next determined whether MKRN2 could ubiquitinate p65 protein. We transfected 293 T cells with expression plasmids encoding p65, MKRN2 and His-tagged ubiquitin. His-tagged proteins were purified by nickel-pull down and polyubiquitination of p65 was detected with a p65 Ab. As show in Fig. 3a, MKRN2 enhanced p65 polyubiquitination in a dose-dependent manner. We next purified MKRN2 protein by immunoprecipitation from 293 T cells transfected with MKRN2 expression plasmid and mixed it *in vitro* with recombinant p65 plus E1, E2 and biotinylated-ubiquitin. MKRN2 could mediate the *in vitro* ubiqutination of p65 protein (Fig. S1), suggesting that MKRN2 could directly target p65 for polyubiquitination. To examine the effects of MKRN2 on p65 protein amounts, we measured p65 in soluble and insoluble nuclear fractions of NIH3T3 cells transfected with FLAG-tagged p65 and MKRN2 expression plasmids. MKRN2 overexpression decreased p65 in the soluble nuclear fraction but increased it in the insoluble nuclear fraction (Fig. 3b). Treatment of these cells with MG132, an inhibitor of proteasomal degradation, resulted in accumulation of p65 in the insoluble nuclear fraction (Fig. 3c). In contrast, p65 in the soluble nuclear fraction, which was decreased by MKRN2 expression, was not restored by MG132 treatment. These data suggest that MKRN2 shuttles p65 from soluble to insoluble nuclear compartments, where p65 is ultimately degraded by the proteasome. To clarify the function of the RING finger domain in p65 ubiquitination and degradation, we generated a MKRN2 mutant lacking a RING finger domain (Fig. 3d). Although ∆RING mutant could bind to p65 (Fig. S2a), this mutant was impaired in its ability to polyubiquitinate p65 (Fig. 3e), indicating the important role of RING finger domain in MKRN2-mediated p65 polyubiquitination. We then transfected NIH3T3 cells with plasmids encoding wild-type or MKRN2-∆RING, together with FLAG-tagged p65. As expected, wild-type MKRN2 decreased soluble p65 and increased insoluble p65 (Fig. 3b,f). MKRN2-∆RING reduced soluble p65 as efficiently as wild-type, suggesting that the RING finger domain-mediated p65 polyubiquitination is not required for intranuclear trafficking of p65 from soluble to insoluble compartments. Indeed, the activity of MKRN2-∆RING to suppress p65-mediated gene activation was partially impaired due to insufficient p65 degradation (Fig. S2b), but not completely abrogated since this mutant retained the activity to sequestrate p65 into discrete intranuclear compartment, in which p65 could be separated from sites of active transcription. Notably, there was much more insoluble p65 in MKRN2-∆RING transfectants than in wild-type transfectants (Fig. 3f). This can be ascribed to reduced p65 degradation in the insoluble fraction, as MKRN2-∆RING is impaired in its ability to polyubiquitinate p65. These data indicate that MKRN2 promotes polyubiquitination and degradation of p65 through its RING finger domain.

### MKRN2 and PDLIM2 synergistically promote p65 ubiquitination and degradation

**Figure 4:**
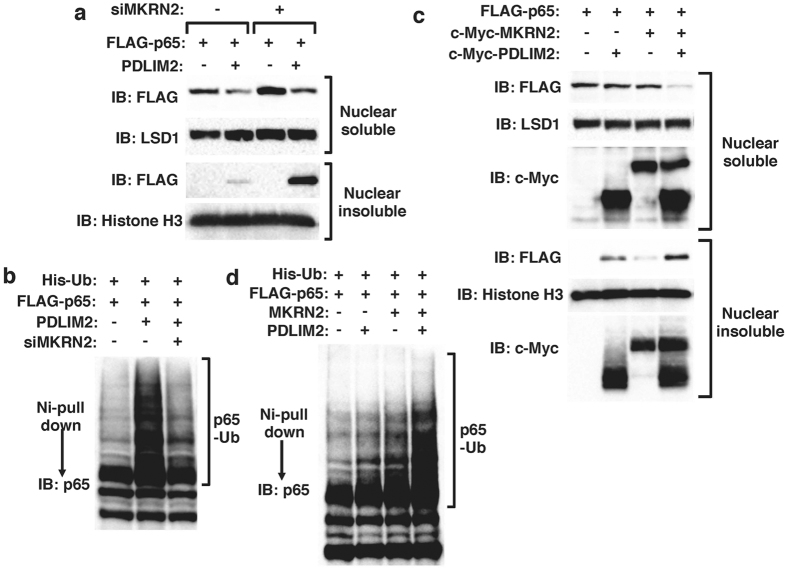
MKRN2 and PDLIM2 synergistically promote p65 ubiquitination. (**a**) Effect of MKRN2 deficiency on soluble and insoluble nuclear p65 in NIH3T3 cells first transfected with control siRNA or MKRN2-specific siRNA, then transfected with plasmids encoding FLAG-p65 or c-Myc-PDLIM2 and analyzed by anti-FLAG antibody. (**b**) Effect of MKRN2 deficiency on ubiquitination of p65 in 293 T cells cotransfected with plasmids encoding His-tagged ubiquitin, p65 and PDLIM2, in the absence or presence of siRNA specific for MKRN2 and analyzed as in Fig. 3a. (**c**) Synergic effect of MKRN2 and PDLIM2 on soluble and insoluble nuclear p65 in NIH3T3 cells transfected with plasmids encoding p65, MKRN2 or PDLIM2 in the indicated combination and analyzed by anti-FLAG antibody. (**d**) Ubiquitination assay for p65 in 293 T cells cotransfected with plasmids encoding His-tagged ubiquitin, p65, MKRN2 or PDLIM2 in the indicated combinations and analyzed as in Fig. 3a.

The effect of MKRN2 on p65 ubiquitination and degradation was quite similar to that of PDLIM2^9^. We therefore examined whether MKRN2 and PDLIM2 work cooperatively to regulate p65 activation. To examine the effects of MKRN2 on PDLIM2-mediated p65 degradation, we first measured p65 levels in soluble and insoluble nuclear fractions of NIH3T3 cells transfected with p65 and PDLIM2 expression plasmids in the absence or presence of MKRN2-specific siRNA to target the endogenous transcripts. MKRN2 knockdown resulted in accumulation of p65 mainly in the insoluble nuclear fraction where p65 degradation takes place (Fig. 4a). Moreover, knockdown of MKRN2 by siRNA, in which MKRN2 mRNA level was reduced to about 43% of that in control cells, impaired PDLIM2-mediated p65 polyubiquitination (Fig. 4b). Since the degradation of polyubiquitinated p65 protein mainly occurred in insoluble nuclear compartment^9^, these data suggest that MKRN2 is necessary for the activity of PDLIM2 to polyubiquitinate and degrade p65 protein in the insoluble nuclear fraction. We next transfected 293 T cells with plasmids encoding FLAG-tagged p65 and c-Myc-tagged PDLIM2 together without or with c-Myc-tagged MKRN2 in a suboptimal condition. While the expression of either PDLIM2 or MKRN2 alone minimally decreased p65 protein level under these conditions, coexpression of PDLIM2 and MKRN2 dramatically decreased p65 protein in the soluble nuclear fraction and increased p65 protein in the insoluble nuclear fraction (Fig. 4c). We then transfected 293 T cells with plasmids encoding FLAG-tagged p65, His-tagged ubiquitin and PDLIM2, together without or with c-Myc-tagged MKRN2 in a suboptimal condition and then examined the effect of MKRN2 on PDLIM2-mediated p65 ubiqutination. The presence of either PDLIM2 or MKRN2 slightly enhanced p65 ubiquitination, whereas the presence of both proteins resulted in a marked induction of p65 polyubiquitination (Fig. 4d). These data suggest that MKRN2 and PDLIM2 synergistically promote polyubiquitination and subsequent degradation of p65.

### Enhanced p65-mediated inflammatory responses accompany MKRN2 deficiency

**Figure 5:**
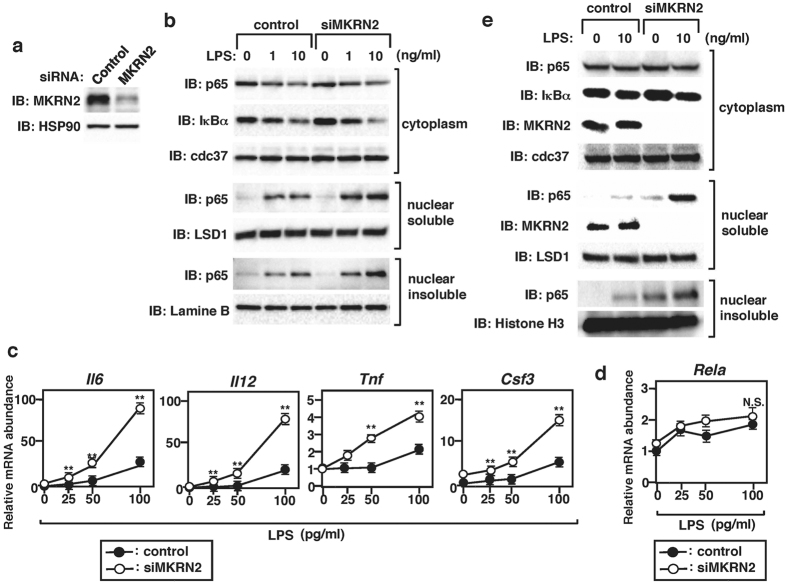
Enhanced p65-mediated inflammatory responses due to MKRN2 deficiency. (**a**) Reduction of MKRN2 protein amount in bone marrow-derived dendritic cells, transfected with siRNA specific for MKRN2, analyzed by immunoblot with anti-MKRN2. Data are representative of three independent experiments. (**b**) Western blot analysis for cytoplasmic and soluble and insoluble nuclear p65 in bone marrow-derived dendritic cells transfected with control or MKRN2-specific siRNA, then left unstimulated or stimulated with the indicated concentration of LPS for 1 h and analyzed by antibodies against the indicated targets. The purity of the fractionations was confirmed by anti-cdc37 (cytoplasm), anti-LSD1 (nuclear soluble) or anti-Lamine B (nuclear insoluble) antibody staining. Western blots are representative of three independent experiments. (**c**) Real-time RT-PCR analysis of LPS-inducible gene expression in bone marrow-derived dendritic cells transfected with control or MKRN2-specific siRNA, then left unstimulated or stimulated with the indicated concentration of LPS for 5 h. (**d**) Real-time RT-PCR analysis of p65 gene expression in bone marrow-derived dendritic cells transfected with control or MKRN2-specific siRNA, then left unstimulated or stimulated with the indicated concentration of LPS for 5 h. (**e**) Western blot analysis for cytoplasmic and soluble and insoluble nuclear p65 in NIH3T3 cells transfected with control or MKRN2-specific siRNA, then left unstimulated or stimulated with LPS (10 ng/ml) for 1 h and analyzed by antibodies against the indicated targets. The purity of the fractionations was confirmed by anti-cdc37 (cytoplasm), anti-LSD1 (nuclear soluble) or anti-Histone H3 (nuclear insoluble) antibody staining. Western blots are representative of three independent experiments.

Finally, we investigated the physiological roles of MKRN2 in the regulation of TLR-mediated p65 activation. We knocked down MKRN2 in bone marrow-derived dendritic cells by using siRNA, which resulted in a substantial reduction of MKRN2 protein amount compared to control cells (Fig. 5a). We then stimulated the cells with LPS and analyzed p65 by immunoblot with the p65 Ab. Specific knockdown of MKRN2 resulted in a substantial increase in nuclear soluble and insoluble p65 protein compared to control cells, but the amounts of cytoplasmic p65 were unaffected by MKRN2 deficiency (Fig. 5b). Notably, LPS-induced degradation of IκBα was comparable between control and MKRN2 knock down cells. These findings indicate that LPS-dependent nuclear translocation of p65 was normal in MKRN2 knock down cells, thereby suggesting that the increased amount of nuclear p65 protein in these cells was due to impaired p65 degradation in the nucleus. Next, we examined TLR-induced proinflammatory cytokine production in MKRN2 knock down dendritic cells and observed a consistent two- to fivefold increase in IL-6, IL-12p40, TNFα and G-CSF transcripts in response to LPS compared to control cells (Fig. 5c). Since C3H-type zinc finger domain-containing proteins have been reported to bind to and regulate the stability of mRNA encoding the target protein^22^,^23^, we further examined p65 mRNA levels in the MKRN2 knock down dendritic cells. As shown in Fig. 5d, p65 mRNA expression was not altered by MKRN2 deficiency, thereby excluding the possibility of an effect of MKRN2 on p65 mRNA stability. We also examined the effect of MKRN2 knockdown in NIH3T3 cells. Although MKRN2 expression was almost completely abrogated in both the cytoplasm and in the nucleus by siRNA, nuclear soluble and insoluble, but not cytoplasmic, p65 was increased in MKRN2 knock down cells (Fig. 5e). These data suggest that MKRN2 inhibits NF-κB-mediated inflammatory responses by controlling nuclear p65 protein levels.

## Discussion

NF-κB activation must be terminated at appropriate time points to prevent excessive inflammation leading to immunopathogy^3^,^4^. It is well known that NF-κB subunits can be inactivated by polyubiquitination and degradation by the proteasome^24^. We have previously reported that PDLIM2 acts as a nuclear ubiquitin E3 ligase for the p65 subunit of NF-κB through its LIM domain, negatively regulating p65-mediated inflammatory responses *in vitro* and *in vivo*^9^. In addition, several ubiquitin E3 ligases targeting p65 for proteasomal degradation have been reported. These include SOCS1 (suppressor of cytokine signaling 1)^25^, COMMD1 (COMM domain containing 1)^26^, PPARγ (peroxisome proliferator activated receptor-γ)^27^ and ING4 (inhibitor of growth protein 4)^28^. In this study, we used yeast two-hybrid screening to isolate MKRN2 based on its ability to associate with PDLIM2 and identified MKRN2 as a novel ubiquitin E3 ligase for p65. We demonstrated that MKRN2 bound to p65 and promoted polyubiquitination and proteasome-dependent degradation of p65 through its RING finger domain, negatively regulating inflammatory responses in dendritic cells. Moreover, we also showed that MKRN2 and PDLIM2 synergistically control p65 polyubiquitination and degradation. It is not unusual that a target protein can be ubiquitinated by multiple ubiquitin E3 ligases. However, it is not well understood whether these ubiquitin E3 ligases independently or cooperatively regulate target protein levels. To date, only a few instances of functional dimerization of ubiquitin E3 ligases have been reported. These include heterodimers of BRCA1-BARD1^16^ and MDM2-MDMX^14^,^15^. In both cases, two ubiquitin E3 ligases synergistically promote ubiquitination and inactivation of target proteins. Although the exact mechanisms by which these ubiquitin E3 ligases work together have not been clarified, several possiblilities have been proposed. For example, it has been shown that BARD1 and MDMX enhance ubiquitn E3 ligase activity of BRCA1 and MDM2 respectively by stabilizing these proteins^14^,^15^,^16^. We therefore checked if PDLIM2 protein levels in dendritic cells might be affected by MKRN2 knock down, but found that they were comparable to those in control cells (**data not shown**). This suggests that MKRN2 and PDLIM2 utilize a different mechanism from other ubiquitin E3 ligases to cooperatively regulate p65 activation, although the exact mechanism is still under investigation. Notably, knockdown of either MKRN2 or PDLIM2 alone resulted in dysregulated p65 activation (Fig. 5b,c,e), suggesting that MKRN2 and PDLIM2 work cooperatively, but cannot fully compensate each other.

Since it is constitutively expressed in the nucleus^7^, PDLIM2 is considered to be a nuclear ubiquitin E3 ligase for p65. In contrast, MKRN2 is located in both the cytoplasm and the nucleus. However, we assume that MKRN2 degrades p65 mainly in the nucleus, since nuclear, but not cytoplasmic, p65 amounts were increased in MKRN2 knock down dendritic cells and fibroblasts compared to control cells (Fig. 5b,e). MKRN2 may not be able to effectively polyubiquitinate p65 in the cytoplasm due to the lack of PDLIM2. Alternatively, as we previously reported^9^, polyubuquitination and degradation of p65 itself may occur only in the nucleus, and not in the cytoplasm, even in the presence of MKRN2.

In addition to its RING finger domain, MKRN2 also has C3H-type zinc finger domains, which are though to be RNA-binding structures. Proteins that have both RING finger and RNA-binding domains, such as a C3H-type zinc finger domain or a KH domain, are classified as RNA-binding E3 ligase family members^29^, and include ROQUIN^22^ and MEX-3A^23^. These proteins bind to the 3′-untranslated region of *Icos* and *HLA-A2* mRNAs, respectively and promote their degradation. Some reports have shown that the RING finger domain is required for mRNA degradation^23^,^30^; however, the exact functional link between unbiquitination and mRNA turnover has not yet been defined. MKRN2 was also reported to negatively regulate phosphatidylinositol 3-kinase (PI3K)/Akt-induced neurogenesis by up-regulating *Gsk-3β* mRNA^12^, although the molecular mechanisms responsible for this activity were not clarified. We therefore checked if p65 mRNA levels might be affected by MKRN2 knock down. However, p65 mRNA abundance in MKRN2 knock down dendritic cells was comparable to that in control cells (Fig. 5d), suggesting that MKRN2 is unlikely to control p65 mRNA levels through its zinc finger domain to inhibit p65 activation.

We have previously shown that PDLIM2 not only acts on p65 protein via its LIM domain to cause ubiquitination but also targets p65 to discrete subnuclear domains, called PML nuclear bodies, through its PDZ domain^9^. The ubiquitinated p65 protein then undergoes 26S proteasome-dependent degradation in these intranuclear compartments. In this study, we demonstrated that MKRN2 promotes polyubiquitination of p65 and also facilitates the transport of p65 from soluble to insoluble nuclear compartments, where p65 is ultimately degraded by the proteasome, although we were unable to demonstrate the colocalization of this insoluble p65 with PML nuclear bodies (Fig. 3b,c). Notably, the RING finger domain of MKRN2 is essential for it to ubiquitinate p65 but is not required for intranuclear trafficking of p65 into insoluble nuclear compartments (Fig. 3f). These effects of MKRN2 on p65 inactivation are quite similar to that of PDLIM2, further supporting their functional cooperation.

Finally, we demonstrate that MKRN2 knock down in dendritic cells results in enhanced expression of proinflammatory cytokines, including IL-6, IL-12, TNFα and G-CSF, indicating that MKRN2 negatively regulates NF-κB-mediated inflammatory responses. Constitutive activation of NF-κB at sites of inflammation is observed in certain human autoimmune and inflammatory diseases, such as rheumatoid arthritis and bronchial asthma^3^,^4^. Thus, the MKRN2-mediated pathway to inhibit p65 activation could be a useful new molecular target for the treatment of autoimmune and inflammatory diseases.

## Materials and Methods

### Expression Vector

The c-Myc-tagged or FLAG-tagged MKRN2 was generated by subcloning of the coding sequence of mouse *Mkrn2* into pCMV-Myc or pCMV-DYKDDDDK (Clontech), respectivly. For the ∆RING-MKRN2 mutant, the cDNA corresponding to amino acids 1–236 plus 292–416 of *Mkrn2* was subcloned into pCMV-Myc. His-tagged PDLIM2, c-Myc-tagged PDLIM2 and FLAG-tagged p65 were previously described^7^,^9^. To generate the plasmid encoding a p65-EGFP fusion protein for imaging experiments, human *RelA* cDNA was inserted in-frame with EGFP into the pCMV-EGFP vector. The ELAM-1 luciferase reporter construct was kindly provided by D. Golenbock. The pGL4.32-Null renilla construct was purchased from Promega and modified.

### Yeast Two-Hybrid Screening

To generate the yeast two-hybrid bait construct, a full length murine *Pdlim2* cDNA was subcloned into the pGBKT7 vector (Clontech). Yeast strain Y2H Gold was transformed with bait plasmid and then mated with yeast strain Y187, which expressed a cDNA library constructed from 18.5 d.p.c. embryos as prey proteins. Positive clones were selected on drop out, X-gal and Aureobasidin A plates, and cDNAs from these clones were retrieved and sequenced. All experiments for yeast two-hybrid screening were conducted according to the manufacturer’s protocol (Clontech).

### Reagents and antibodies

LPS (from Salmonella enterica; L-2262) was purchased from Sigma. Murine GM-CSF was from R&D systems. Ubiquitilation kit (BML-UW9920) and recombinant p65 (BML-UW9995) were purchased from Enzo Life Sciences. Anti-DYKDDDDK (NU01102) antibody was purchased from Nacalai USA and used as anti-FLAG antibody. Anti-p65 (sc-372), IκBα (sc-371), HSP90 (sc-7947), Lamin B (sc-6217) and Omni-probe (sc-499; used as anti-His) antibodies were purchased from Santa Cruz Biotechnology. Anti-LSD1 (#2184), anti-cdc37 (#3604) and anti-Histone H3 (#4499) antibodies were purchased from Cell Signaling Technology. Anti-MKRN2 (#72055) antibody was purchased from Abcam. Anti-c-Myc (M047–3) antibody, anti-c-Myc antibody-conjugated agarose (M047-8) and DYKDDDDK-tagged protein PURIFICATION GEL (#3328) (used as anti-FLAG antibody-conjugated agarose) were purchased from MBL. 3xFLAG peptide (F4799) was purchased from Sigma-Aldrich. The secondary antibodies HRP-goat anti-rabbit IgG (#656120) was purchased from Zymed and HRP-conjugated sheep anti-mouse IgG (NA931) was purchased from GE Healthcare. HRP-Conjugated Streptavidin (#554066) was purchased from BD Pharmingen. Alexa Fluor 647-conjugated goat anti-mouse IgG1 (A21241) was from Thermo Fisher Scientific.

### Cells, transfection, reporter assay

Mouse embryonic fibroblasts (MEF) prepared from 13.5 d.p.c. embryos, HeLa and 293 T cells were cultured in DMEM supplemented with 10% fetal bovine serum (FBS). NIH3T3 cells were cultured in DMEM supplemented with 10% calf serum. RAW264.7 cells were cultured in RPMI1640 supplemented with 10% FBS. Bone marrow derived dendritic cells were obtained by culture of bone marrow cells for 5 days with murine GM-CSF (10 ng/ml) in RPMI1640 supplemented with 10% FBS (Fig. 5a–d). CD4^+^, CD8^+^, CD19^+^ and CD11c^+^ cells were purified from the spleen with MACS columns (Miltenyi Biotech). For transfections, cells were transiently transfected with Effectene (QIAGEN). For the reporter assay, NIH3T3 cells (Fig. 2b,c) were transfected with the ELΑΜ−1 luciferase and pGL4-Null renilla constructs and expression plasmids encoding p65 without or with MKRN2. Alternatively, MEF (Fig. 2a) were transfected without or with the MKRN2 vector, then stimulated for 5 hr with LPS. The total amount of transfected DNA in each case was kept constant by supplementing with control plasmids. Luciferase activity was measured according to the manufacturer’s protocol in the Dual Luciferase Reporter System (Promega).

### Subcellular fractionation, immunoprecipitation and immunoblot analysis

All lysis buffers used for immunoblot analysis contained a proteinase inhibitor ‘cocktail’ (complete mini; Roche). Cytoplasmic, nuclear soluble, and nuclear insoluble extracts were prepared as previously described^9^. The purity of the obtained fractions was confirmed using anti-cdc37 (for cytoplasmic fraction), anti-LSD1 (for nuclear soluble fraction), and anti-Histone H3 (for nuclear insoluble fraction). Whole cell extracts were prepared by lysing cells in 50 mM Tris pH 8.0, 0.5% NP-40, 5 mM EDTA, 50 mM NaCl, 50 mM NaF (Fig. 1b). For immunoprecipitation from 293 T cells (Figs 1d and 2d), cells were lysed in 0.5% NP-40, 250 mM NaCl, 10 mM Tris pH 8.0, incubated with anti-c-Myc antibody-conjugated agarose beads (MBL), washed four times and subjected to immunoblot analysis with the indicated antibodies.

### Ubiquitination assay

For *in vivo* ubiquitination assay, 293 T cells were transfected with expression plasmids encoding FLAG-tagged p65, His-tagged ubiquitin and c-Myc-MKRN2 and/or PDLIM2. His-tagged proteins were then purified as previously described^31^. Briefly, transfected cells were extracted under denaturing condition with a buffer containing 6 M guanidium-HCl. Extracts were incubated with Ni-Sepharose beads (GE HealthCare) for 2.5 hr and then washed with buffer containing 25 mM Tris pH 6.8, 20 mM imidazole. Purified proteins were subjected to immunoblot with anti-p65. *In vitro* ubiquitination assay was performed using Ubiquitilation kit (Enzo Life Sciences) according to the manufacturer’s protocol. For *in vitro* ubiquitination assay for p65, MKRN2 proteins, immunoprecipitated with anti-DYKDDDDK antibody-conjugated agarose from 293 T cells transfected with a FLAG-tagged MKRN2, then eluted by 3xFLAG peptide, were incubated 2 hr with E1, UbcH5c, biotin-ubiquitin and recombinant p65. Ubiquitinated p65 was detected by immunoprecipitation with anti-p65 and immunoblot with HRP-Conjugated Streptavidin.

### Immunofluorescence

HeLa cells were seeded onto a 35-mm glass-bottom culture dish (MatTek) and transfected with expression plasmids encoding c-Myc-MKRN2 and EGFP-tagged p65 using Lipofectamine and PLUS reagent (Thermo Fisher Scientific). The next day, cells were fixed for 20 min with 4% paraformaldehyde and were made permeable by treatment for 10 min with 0.5% Triton X-100 on ice. After blocking with 3 mg/mL bovine serum albumin for 1hr, cells were incubated for 30 min with the Myc antibody (1:1000 dilution) and then for 30 min with Alexa Fluor 647-conjugated goat anti-mouse IgG1 (1:100 dilution).

### Microscopy

Imaging by fluorescence microscopy^32^ was performed on an inverted microscope (IX-83, Olympus) with a 100 × objective (PlanApo 100 × NA1.45 Oil, Olympus) using a HILO and TIRF illuminator (Cell TIRF, Olympus). Solid-state laser (488 nm, 20 mW, Sapphire 488-20-PS, Coherent) and CoolLED fluorescence light source (635 nm, Molecular Devices) were used for fluorescence excitation. Images were captured with two back-thinned electro multiplier charge coupled device cameras (EMCCD, C9100-13, Hamamatsu Photonics). Images were recorded with AQUACOSMOS software (Hamamatsu Photonics) and analyzed using ImageConverter software (Olympus Software Technology)^33^.

### Small interfering RNA (siRNA)

Lipofectamine RNAiMAX (Invitrogen) was used for the transfection of siRNA (Figs 2c,4a,b and 5c–e). Alternatively, a mouse dendritic cell Nucleofector kit with Nucleofector I was used for transfection (Fig. 5a,b). The following siRNA were purchased from Invitrogen (Stealth RNAi); murine *Mkrn2*, 5′-AGACCAGTGAGATTGCGGTCTCTGA-3′; human *MKPN2*, 5′- TTGCCTTGCTCAAAGTATTTACAGG -3′; and control siRNA (12935-300).

### Real-time reverse transcriptase-polymerase chain reaction (RT-PCR) analysis

Total RNA was prepared using an RNAeasy micro kit (Qiagen) and cDNA was generated using a PrimeScript RT reagent kit (Takara Bio Inc.). Quantitative real-time PCR analyses were performed using a StepOnePlus (Applied Biosystems). The primer sets and probes for mouse *Il-6* (Mm00446190), *Il-12* (Mm00434174), *Tnf*α (Mm00443258), *Csf3* (Mm00438334), and 18S rRNA (4319413E) from TaqMan Gene Expression Assay (Applied Biosystems) were used for the reactions. To analyze mouse and human MKRN2 expression, the following primer pairs were used; mouse *Mkrn2*, 5′-GTCCTGCACCCAACCCTTC-3′ and 5′-CACCAGCGTCTTCTTCTCCC-3′; human *MKRN2*, 5′-AGGAAGTCAGTGCCTATTCTCA-3′ and 5′-TGGTCATATCTGCACCGAGTT-3′ (Invitrogen). Data were normalized to the amounts of 18S rRNA.

### Mice

All mice used were between 7 to 8 weeks of age and were purchased from CREA Japan, Inc. All experiments were approved by the RIKEN Yokohama Campus Animal Use Committee, and performed in accordance with the committee’s guidelines.

### Statistical analysis

All of the experiments were repeated at least three times. Differences were analyzed by Student’s t-test. Data are presented as the mean values ± the standard deviation of the mean (SD).

## Additional Information

**How to cite this article:** Shin, C. *et al*. MKRN2 is a novel ubiquitin E3 ligase for the p65 subunit of NF-κB and negatively regulates inflammatory responses. *Sci. Rep.* **7**, 46097; doi: 10.1038/srep46097 (2017).

**Publisher's note:** Springer Nature remains neutral with regard to jurisdictional claims in published maps and institutional affiliations.

## Supplementary Material

Supplementary Information

## References

[b1] Hayden M. S. & Ghosh S. Signaling to NF-κB. Genes & Dev. 18, 2195–2224 (2004). 15371334 10.1101/gad.1228704

[b2] Takeuchi O. & Akira S. Pattern recognition receptors and inflammation. Cell. 140, 805–820 (2010). 20303872 10.1016/j.cell.2010.01.022

[b3] Gregersen P. K. & Olsson L. M. Recent advances in the genetics of autoimmune diseases. Annu. Rev. Immunol. 27, 363–391 (2009). 19302045 10.1146/annurev.immunol.021908.132653 PMC2992886

[b4] Sun S. C., Chang J. H. & Jin J. Regulation of nuclear factor-κB in autoimmunity. Trends Immunol. 34, 282–289 (2013). 23434408 10.1016/j.it.2013.01.004 PMC3664242

[b5] Kondo T., Kawai T. & Akira S. Dissecting negative regulation of Toll-like receptor signaling. Trends Immunol. 33, 449–458 (2012). 22721918 10.1016/j.it.2012.05.002

[b6] Torrado M., Senatorov V. V., Trivedi R., Fariss R. N. & Tomarev S. I. Pdlim2, a novel PDZ-LIM domain protein, interacts with α-actinins and filamin A. Invest. Ophthalmol. Vis. Sci. 45, 3955–3963 (2004). 15505042 10.1167/iovs.04-0721

[b7] Tanaka T., Soriano M. A. & Grusby M. J. SLIM is a nuclear ubiquitin E3 ligase that negatively regulates STAT signaling. Immunity 22, 729–736 (2005). 15963787 10.1016/j.immuni.2005.04.008

[b8] Loughran G. . Mystique is a new insulin-like growth factor-I-regulated PDZ-LIM domain protein that promotes cell attachment and migration and suppresses Anchorage-independent growth. Mol Biol Cell. 16, 1811–1822 (2005). 15659642 10.1091/mbc.E04-12-1052 PMC1073663

[b9] Tanaka T., Grusby M. J. & Kaisho T. PDLIM2-mediated termination of transcription factor NF-κB activation by intranuclear sequestration and degradation of the p65 subunit. Nat. Immunol. 8, 584–591 (2007). 17468759 10.1038/ni1464

[b10] te Velthuis A. J. & Bagowski C. P. PDZ and LIM domain-encoding genes: molecular interactions and their role in development. ScientificWorldJournal 7, 1470–1492 (2007). 17767364 10.1100/tsw.2007.232 PMC5901285

[b11] Zhang Q. H. . Cloning and functional analysis of cDNAs with open reading frames for 300 previously undefined genes expressed in CD34+ hematopoietic stem/progenitor cells. Genome Res. 10, 1546–1560 (2000). 11042152 10.1101/gr.140200 PMC310934

[b12] Yang P. H. . Makorin-2 is a neurogenesis inhibitor downstream of phosphatidylinositol 3-kinase/Akt (PI3K/Akt) signal. J. Biol. Chem. 283, 8486–8495 (2008). 18198183 10.1074/jbc.M704768200 PMC2417189

[b13] Deshaies R. J. & Joazeiro C. A. RING domain E3 ubiquitin ligases. Annu. Rev. Biochem. 78, 399–434 (2009). 19489725 10.1146/annurev.biochem.78.101807.093809

[b14] Tanimura S. . MDM2 interacts with MDMX through their RING finger domains. FEBS Lett. 447, 5–9 (1999). 10218570 10.1016/s0014-5793(99)00254-9

[b15] Wang X. & Jiang X. Mdm2 and MdmX partner to regulate p53. FEBS Lett. 586, 1390–1396 (2012). 22673503 10.1016/j.febslet.2012.02.049

[b16] Hashizume R. . The RING heterodimer BRCA1-BARD1 is a ubiquitin ligase inactivated by a breast cancer-derived mutation. J. Biol. Chem. 276, 14537–14540 (2001). 11278247 10.1074/jbc.C000881200

[b17] Gray T. A. . The ancient source of a distinct gene family encoding proteins featuring RING and C_3_H zinc-finger motifs with abundant expression in developing brain and nervous system. Genomics 66, 76086 (2000). 10.1006/geno.2000.6199 10843807

[b18] Böhne A. . The vertebrate makorin ubiquitin ligase gene family has been shaped by large-scale duplication and retroposition from an ancestral gonad-specific, maternal-effect gene. BMC Genomics 11, 721 (2010). 21172006 10.1186/1471-2164-11-721 PMC3022923

[b19] Kim J. H. . Ubiquitin ligase MKRN1 modulates telomere length homeostasis through a proteolysis of hTERT. Genes & Dev. 19, 776–781 (2005). 15805468 10.1101/gad.1289405 PMC1074314

[b20] Lee E. W. . Differential regulation of p53 and p21 by MKRN1 E3 ligase controls cell cycle arrest and apoptosis. EMBO J. 28, 2100–2113 (2009). 19536131 10.1038/emboj.2009.164 PMC2718286

[b21] Lee K. Y. . Ubiquitous expression of MAKORIN-2 in normal and malignant hematopoietic cells and its growth promoting activity. PLoS One 9, e92706 (2014). 24675897 10.1371/journal.pone.0092706 PMC3968021

[b22] Yu D. . Roquin represses autoimmunity by limiting inducible T-cell co-stimulator messenger RNA. Nature 450, 299–303 (2007). 18172933 10.1038/nature06253

[b23] Cano F. . The RNA-binding E3 ubiquitin ligase MEX-3C links ubiquitination with MHC-I mRNA degradation. EMBO J. 31, 3596–606 (2012). 22863774 10.1038/emboj.2012.218 PMC3433784

[b24] Collins P. E., Mitxitorena I. & Carmody R. J. The Ubiquitination of NF-κB Subunits in the Control of Transcription. Cells 5, 23 (2016) 10.3390/cells5020023 PMC4931672 27187478

[b25] Ryo A. . Regulation of NF-kappaB signaling by Pin1-dependent prolyl isomerization and ubiquitin-mediated proteolysis of p65/RelA. Mol. Cell 12, 1413–1426 (2003). 14690596 10.1016/s1097-2765(03)00490-8

[b26] Maine G. N., Mao X., Komarck C. M. & Burstein E. COMMD1 promotes the ubiquitination of NF-κB subunits through a cullin-containing ubiquitin ligase. EMBO J 26, 436–447 (2007). 17183367 10.1038/sj.emboj.7601489 PMC1783443

[b27] Hou Y. . Inhibitor of growth 4 induces NFκB/p65 ubiquitin-dependent degradation. Oncogene 33, 1997–2003 (2014). 23624912 10.1038/onc.2013.135

[b28] Hou Y., Moreau F. & Chadee K. PPARγ is an E3 ligase that induces the degradation of NFκB/p65. Nat. Commun. 3, 1300 (2012). 23250430 10.1038/ncomms2270

[b29] Cano F., Miranda-Saavedra D. & Lehner P. J. RNA-binding E3 ubiquitin ligases: novel players in nucleic acid regulation. Biochem. Soc. Trans. 38, 1621–1626 (2010). 21118137 10.1042/BST0381621

[b30] Laroia G., Sarkar B. & Schneider R. J. Ubiquitin-dependent mechanism regulates rapid turnover of AU-rich cytokine mRNAs. Proc. Natl. Acad. USA 99, 1842–1846 (2002). 10.1073/pnas.042575699 PMC122281 11842200

[b31] Campanero M. R. & Flemington E. K. Regulation of E2F through ubiquitin-proteasome-dependent degradation: stabilization by the pRB tumor suppressor protein. Proc. Natl. Acad. USA 94, 2221–2226 (1997). 10.1073/pnas.94.6.2221 PMC20068 9122175

[b32] Tokunaga M., Imamoto N. & Sakata-Sogawa K. Highly inclined thin illumination enables clear single-molecule imaging in cells. Nat. Methods 5, 159–161 (2008). 18176568 10.1038/nmeth1171

[b33] Ito Y., Sakata-Sogawa K. & Tokunaga M. A Facile Preparation of Glass-supported Lipid Bilayers for Analyzing Molecular Dynamics. Anal. Sci. 30, 1103–1106 (2014). 25492457 10.2116/analsci.30.1103

