# Supplementary material for: MKRN2 is a novel ubiquitin E3 ligase for the p65 subunit of NF-κB and negatively regulates inflammatory responses
Source: Sci Rep. 2017 Apr 5;7:46097. doi: 10.1038/srep46097 (PMC5380948; doi:10.1038/srep46097)
Supplement: Supplementary Information [file srep46097-s1.pdf]

Supplementary Information

MKRN2 is a novel ubiquitin E3 ligase for the p65 subunit of NF-κB and negatively regulates inflammatory responses

Chanyoung Shin[1,2], Yuma Ito[2], Shota Ichikawa[2], Makio Tokunaga[2], Kumiko Sakata-Sogawa[2] & Takashi Tanaka[1*]

[1]Laboratory for Inflammatory Regulation, RIKEN Center for Integrative Medical Sciences (IMS), RIKEN Research Center for Allergy and Immunology (RCAI), Yokohama, Kanagawa 230-0045, Japan

[2]School of Life Science and Technology, Tokyo Institute of Technology, Yokohama, Kanagawa 226-8501, Japan

[*]**Corresponding author:** E-mail address: takashi.tanaka@riken.jp

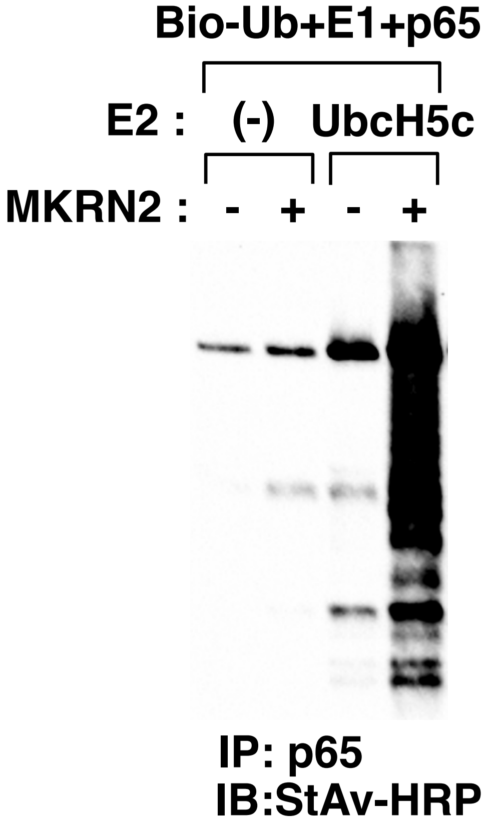

Supplementary Fig. 1. MKRN2 is a ubiquitin E3 ligase targeting p65.

MKRN2 promotes ubiqutination of p65 in vitro. MKRN2 proteins, immunoprecipitated with anti-FLAG from 293T cells transfected with a FLAG-tagged MKRN2, then eluted by 3xFLAG peptide, were incubated in vitro with ubiquitin components as indicated including recombinant p65. Ubiquitinated p65 was detected by immunoprecipitation with anti-p65 and immunoblot with HRP-Conjugated Streptavidin.

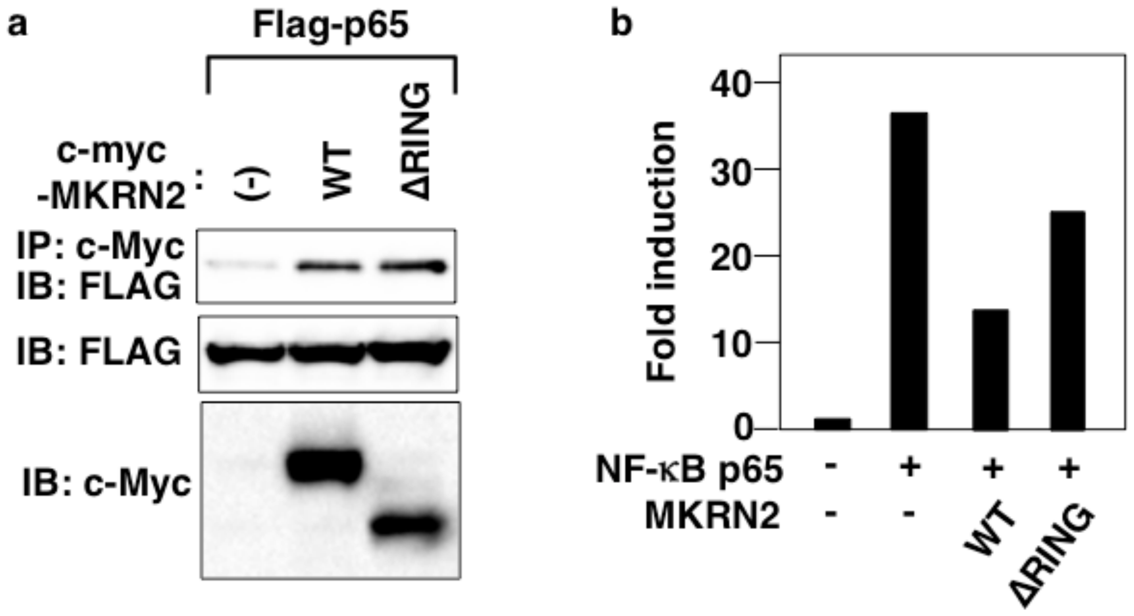

Supplementary Fig. 2. MKRN2 mutant lacking RING finger domain binds to p65 and suppresses p65 transactivation.

(**a**) 293T cells were transfected with a FLAG-tagged p65 expression plasmid along with or without c-Myc-tagged MKRN2 or the MKRN2 mutant lacking the RING finger domain (ΔRING). Whole cell extracts were immunoprecipitated with anti-c-Myc, and immunoblotted with anti-Flag. Western blots are representative of at least three independent experiments. (**b**) Luciferase activity in MEF cells transfected with an ELAM-1-luc with or without plasmids encoding p65 in the absence or presence of expression plasmids encoding wild-type or ΔRING MKRN2. Data are representative of at least three independent experiments.
